# Supplementary material for: A hidden web of policy influence: The pharmaceutical industry’s engagement with UK’s All-Party Parliamentary Groups
Source: PLoS One. 2021 Jun 24;16(6):e0252551. doi: 10.1371/journal.pone.0252551 (PMC8224875; doi:10.1371/journal.pone.0252551)
Supplement: S1 Table — (DOCX) [file pone.0252551.s001.docx]

**S1 Table. Number and value of the pharmaceutical industry’s direct financial and in-kind payments**

| Company | Financial and in-kind payments | | In-kind payments | | Financial payments | |
| --- | --- | --- | --- | --- | --- | --- |
|  | Total value (%) - £ | Number of payments (%) | Value of payments (%) - £ | Total number (%) | Value of payments (%) - £ | Number of payments (%) |
| Pfizer | 76,643.17 (8.93) | 10 (7.75) | 47,426 (9.23) | 6 (8.45) | 29,217.17 (8.47) | 4 (6.9) |
| Bristol-Myers Squibb | 75,734.48 (8.82) | 10 (7.75) | 34,852.48 (6.78) | 4 (5.63) | 40,882 (11.85) | 6 (10.34) |
| Gilead | 68,475.48 (7.97) | 7 (5.43) | 9,947.86 (1.94) | 1 (1.41) | 58,527.62 (16.97) | 6 (10.34) |
| Novartis | 64,038.14 (7.46) | 14* (10.85) | 49,376.56 (9.61) | 12 (16.9) | 14,661.58 (4.25) | 2 (3.45) |
| Sanofi Pasteur MSD | 49,270.7 (5.74) | 7 (5.43) | 33,034.86 (6.43) | 5 (7.04) | 16,235.84 (4.71) | 2 (3.45) |
| Roche | 44,172.87 (5.14) | 6 (4.65) | 29,689.7 (5.78) | 4 (5.63) | 14,483.17 (4.2) | 2 (3.45) |
| MSD | 43,615.64 (5.08) | 5 (3.88) | 27,291.64 (5.31) | 3 (4.23) | 16,324 (4.73) | 2 (3.45) |
| Abbvie | 39,913.83 (4.65) | 6 (4.65) | 18,006.97 (3.5) | 2 (2.82) | 21,906.86 (6.35) | 4 (6.9) |
| Merck | 36,272.8 (4.22) | 5 (3.88) | 14,657.37 (2.85) | 2 (2.82) | 21,615.43 (6.27) | 3 (5.17) |
| Napp Pharmaceuticals | 36,195.64 (4.22) | 4 (3.1) | 27,291.64 (5.31) | 3 (4.23) | 8,904 (2.58) | 1 (1.72) |
| Grünenthal | 34,460.27 (4.01) | 4 (3.1) | 18,224.43 (3.55) | 2 (2.82) | 16,235.84 (4.71) | 2 (3.45) |
| Bayer | 32,250.5 (3.76) | 1 (.78) | 32,250.5 (6.28) | 1 (1.41) | - | - |
| GlaxoSmithKline | 31,686.84 (3.69) | 4 (3.1) | 31,686.84 (6.17) | 4 (5.63) | - | - |
| Janssen | 31,273 (3.64) | 4 (3.1) | 23,853 (4.64) | 3 (4.23) | 7,420 (2.15) | 1 (1.72) |
| Novo Nordisk | 25,852.88 (3.01) | 4 (3.1) | 25,852.88 (5.03) | 4 (5.63) | - | - |
| AstraZeneca | 24,132.64 (2.81) | 4 (3.1) | 21,332.64 (4.15) | 3 (4.23) | 2,800 (.81) | 1 (1.72) |
| Celgene | 19,853.78 (2.31) | 5 (3.88) | 8,067.92 (1.57) | 2 (2.82) | 11,785.86 (3.42) | 3 (5.17) |
| Lundbeck | 18,351.88 (2.14) | 2 (1.55) | 18,351.88 (3.57) | 2 (2.82) | - | - |
| Takeda | 18,351.88 (2.14) | 2 (1.55) | 18,351.88 (3.57) | 2 (2.82) | - | - |
| Boehringer Ingelheim | 16,029.3 (1.87) | 4 (3.1) | 10,196.43 (1.98) | 2 (2.82) | 5,832.86 (1.69) | 2 (3.45) |
| Lilly | 14,440.69 (1.68) | 4 (3.1) | 2,979.92 (.58) | 1 (1.41) | 11,460.77 (3.32) | 3 (5.17) |
| Sanofi† | 13,156.83 (1.53) | 3 (2.33) | 8,068.83 (1.57) | 2 (8.2) | 5,088 (1.48) | 1 (1.72) |
| Otsuka Pharmaceuticals | 10,621.26 (1.24) | 2 (1.55) | - | - | 10,621.29 (3.08) | 2 (3.45) |
| ViiV Healthcare | 7,556.01 (.88) | 1 (.78) | - | - | 7,556.01 (2.19) | 1 (1.72) |
| Vertex Pharmaceuticals | 7,346.53 (.86) | 1 (.78) | - | - | 7,346.53 (2.13) | 1 (1.72) |
| Dermal | 4,967.44 (.58) | 3 (2.33) | - | - | 4,967.44 (1.44) | 3 (5.17) |
| Amgen | 4,633.52 (.54) | 2 (1.55) | 2,979.92 (.58) | 1 (1.41) | 1,653.6 (.48) | 1 (1.72) |
| LEO Pharma | 4,352.17 (.51) | 2 (1.55) | - | - | 4,352.17 (1.26) | 2 (3.45) |
| Derma UK | 3,344.15 (.39) | 2 (1.55) | - | - | 3,344.15 (.97) | 2 (3.45) |
| Almirall | 1,653.6 (.19) | 1 (.78) | - | - | 1,653.6 (.48) | 1 (1.72) |
| Total | 858,647.95 | 129 | 513,772.14 | 71 | 344,875.81 | 58 |

* 4 Novartis benefits in kind had no value

† Sanofi Pasteur included in Sanofi
