# Supplementary material for: A hidden web of policy influence: The pharmaceutical industry’s engagement with UK’s All-Party Parliamentary Groups
Source: PLoS One. 2021 Jun 24;16(6):e0252551. doi: 10.1371/journal.pone.0252551 (PMC8224875; doi:10.1371/journal.pone.0252551)
Supplement: S2 Table — (DOCX) [file pone.0252551.s002.docx]

**S2 Table. Number and value of indirect payments from pharmaceutical companies**

| **Company** | **Number of payments (%*)** | **Number of payments with value (%*)** | **Total value** of payments (%*) - £** |
| --- | --- | --- | --- |
| Novartis | 10 (14.49) | 6 (20) | 89,008.17 (25.24) |
| Bayer | 14 (20.29) | 6 (20) | 62,096.01 (17.61) |
| Pfizer-BMS Alliance | 12 (17.39) | 5 (16.67) | 52,953.24 (15.01) |
| Bluebird Bio | 4 (5.8) | 4 (13.33) | 28,831.13 (8.17) |
| Novo Nordisk | 1 (1.45) | 1 (3.33) | 22,765.61 (6.45) |
| Janssen | 1 (1.45) | 1 (3.33) | 22,765.61 (6.45) |
| Gedeon Richter | 2 (2.9) | 2 (6.67) | 20,986.31 (5.95) |
| Lundbeck | 5 (7.24) | 1 (3.33) | 16,695.53 (4.73) |
| Leo Pharmaceuticals | 3 (4.35) | 1 (3.33) | 16,333.05 (4.63) |
| MSD | 1 (1.45) | 1 (3.33) | 14,625.25 (4.15) |
| Daiichi-Sankyo | 2 (2.9) | 2 (6.67) | 5,637.93 (1.6) |
| Boehringer Ingelheim | 6 (8.82) | - | - |
| Pfizer | 1 (1.45) | - | - |
| Roche | 3 (4.35) | - | - |
| Sanofi | 2 (2.9) | - | - |
| Genus Pharmaceuticals | 1 (1.45) | - | - |
| Total | 69 | 30 | 352,697.86 |

*Percentages are the number/value of payments from each company as a percentage of the total number/value from all pharmaceutical companies

**It is important to note that values of indirect payments were calculated as if all contributors to a given payment provided an equal financial contribution, Therefore, these values are approximate.
