# Supplementary material for: A hidden web of policy influence: The pharmaceutical industry’s engagement with UK’s All-Party Parliamentary Groups
Source: PLoS One. 2021 Jun 24;16(6):e0252551. doi: 10.1371/journal.pone.0252551 (PMC8224875; doi:10.1371/journal.pone.0252551)
Supplement: S3 Table — (DOCX) [file pone.0252551.s003.docx]

**S3 Table. Total number and value of direct and indirect payments from pharmaceutical companies at the recipient level**

| **APPG** | **Total direct and indirect funding received - £** | **Total direct and indirect funding received - n*** | **Total direct and indirect funding received with value - n** | **Total direct and indirect funding received from pharma - £** | **Total direct and indirect funding received from pharma - n** | **Total direct and indirect funding received with value from pharma - n** |
| --- | --- | --- | --- | --- | --- | --- |
| Health | 1,017,516.98 | 108 | 104 | 414,921.47 (40.78) | 47 (43.52) | 47 (45.19) |
| Cancer | 442,318.21 | 54 | 50 | 252,557.67 (57.18) | 45 (83.33) | 45 (90) |
| Sickle Cell and Thalassaemia | 122,527.46 | 16 | 8 | 122,527.46 (100) | 16 (100) | 8 (100) |
| Thrombosis | 224,094.40 | 9 | 5 | 85,330.28 (38.08) | 7 (77.78) | 3 (60) |
| HIV and AIDS | 329,525.96 | 42 | 41 | 66,083.63 (20.05) | 7 (16.67) | 7 (17.07) |
| Obesity | 94,763.94 | 7 | 3 | 45,531.23 (48.05) | 1 (14.29) | 1 (33.33) |
| Atrial Fibrillation | 51,935.62 | 8 | 4 | 42,547.19 (81.92) | 7 (87.5) | 3 (75) |
| Women's Health | 71,223.12 | 3 | 3 | 35,611.56 (50) | 3 (100) | 3 (100) |
| Eye Health and Visual Impairment | 32,250.50 | 9 | 1 | 32,250.5 (100) | 1 (11.11) | 1 (100) |
| Liver Health | 59,906.00 | 14 | 6 | 29,843.59 (49.82) | 7 (50) | 3 (50) |
| Skin | 222,779.70 | 27 | 23 | 23,437.37 (10.52) | 13 (48.15) | 13 (56.52) |
| Tuberculosis | 58,650.74 | 19 | 9 | 19,114.06 (32.59) | 5 (26.32) | 4 (44.44) |
| Sexual and Reproductive Health | 115,768.08 | 16 | 11 | 9,142.77 (7.9) | 1 (6.25) | 1 (9.09) |
| Alcohol Harm | 51,264.61 | 8 | 4 | 16,695.53 (32.57) | 5 (62.5) | 1 (25) |
| Malaria and Neglected Tropical Diseases | 270,418.00 | 35 | 32 | 8,250.50 (3.05) | 1 (2.86) | 1 (3.13) |
| Diabetes | 20,575.70 | 11 | 7 | 7,501.00 (34.46) | 2 (18.18) | 2 (28.57) |
| Total | 3,185,519.01 | 386 | 311 | 1,211,345.81 (38.03) | 168 (43.52) | 143 (45.98) |

** Payments supported by more than one pharmaceutical company are counted as one, i.e. if an indirect payment has at least one pharma company behind it, it is counted as a pharma-sponsored payment with the relative value provided by pharmaceutical companies*
