# Supplementary material for: A hidden web of policy influence: The pharmaceutical industry’s engagement with UK’s All-Party Parliamentary Groups
Source: PLoS One. 2021 Jun 24;16(6):e0252551. doi: 10.1371/journal.pone.0252551 (PMC8224875; doi:10.1371/journal.pone.0252551)
Supplement: S4 Table — (DOCX) [file pone.0252551.s004.docx]

**S4 Table. Total number and value of direct and indirect payments from pharmaceutical companies at the donor level**

| **Pharmaceutical company** | **Total payments – n (%)*** | **Total payments with value – n (%)*** | **Total value of all payments - £ (%)*** |
| --- | --- | --- | --- |
| Novartis | 24 (12.12) | 16 (10.32) | 153,046.31 (12.54) |
| Bayer | 15 (7.58) | 7 (4.52) | 94,346.5 (7.79) |
| Pfizer | 11 (5.56) | 10 (6.45) | 76,643.17 (6.28) |
| Bristol-Myers Squibb | 10 (5.05) | 10 (6.45) | 75,734.48 (6.21) |
| Gilead | 7 (3.54) | 7 (4.52) | 68,475.48 (5.61) |
| MSD | 6 (3.03) | 6 (3.87) | 58,240.89 (4.77) |
| Janssen | 5 (2.53) | 5 (3.23) | 54,039 (4.43) |
| Pfizer-BMS Alliance | 12 (6.06) | 5 (3.23) | 52,953.24 (4.34) |
| Sanofi Pasteur MSD | 7 (3.54) | 7 (4.52) | 49,270.7 (4.04) |
| Novo Nordisk | 5 (2.53) | 5 (3.23) | 48,618.49 (3.98) |
| Roche | 10 (5.05) | 6 (3.87) | 44,172.87 (3.62) |
| Abbvie | 6 (3.03) | 6 (3.87) | 39,913.83 (3.27) |
| Merck | 5 (2.53) | 5 (3.23) | 36,272.8 (2.97) |
| Napp Pharmaceuticals | 4 (2.02) | 4 (2.58) | 36,272.8 (2.97) |
| Lundbeck | 7 (3.54) | 3 (1.94) | 35,047.41 (2.87) |
| Grünenthal | 4 (2.02) | 4 (2.58) | 34,460.27 (2.82) |
| GlaxoSmithKline | 4 (2.02) | 4 (2.58) | 31,686.84 (2.6) |
| Bluebird Bio | 4 (2.02) | 4 (2.58) | 28,831.13 (2.36) |
| AstraZeneca | 4 (2.02) | 4 (2.58) | 24,132.64 (1.98) |
| Gedeon Richter | 2 (1.01) | 2 (1.29) | 20,986.31 (1.72) |
| LEO Pharma | 5 (2.53) | 3 (1.94) | 20,685.22 (1.69) |
| Celgene | 5 (2.53) | 5 (3.23) | 19,853.78 (1.63) |
| Takeda | 2 (1.01) | 2 (1.29) | 18,351.88 (1.5) |
| Boehringer Ingelheim | 10 (5.05) | 4 (2.58) | 16,029.3 (1.31) |
| Lilly | 4 (2.02) | 4 (2.58) | 14,440.69 (1.18) |
| Sanofi | 5 (2.53) | 3 (1.94) | 13,156.82 (1.08) |
| Otsuka Pharmaceuticals | 2 (1.01) | 2 (1.29) | 10,621.29 (.87) |
| ViiV Healthcare | 1 (.51) | 1 (.65) | 7,556.01 (.62) |
| Vertex Pharmaceuticals | 1 (.51) | 1 (.65) | 7,346.53 (.6) |
| Daiichi-Sankyo | 2 (1.01) | 2 (1.29) | 5,637.93 (.46) |
| Dermal | 3 (1.52) | 3 (1.94) | 4,967.44 (.41) |
| Amgen | 2 (1.01) | 2 (1.29) | 4,633.52 (.38) |
| Derma UK | 2 (1.01) | 2 (1.29) | 3,344.15 (.27) |
| Almirral | 1 (.51) | 1 (.65) | 1,653.6 (.14) |
| Genus Pharmaceuticals | 1 (.51) | 0 | - |
| Totals | 198 | 155 | 1,211,345.23 |

*Percentages are the number/value of payments from each company as a proportion of the number/value of payments from all pharmaceutical companies
