# Supplementary material for: A hidden web of policy influence: The pharmaceutical industry’s engagement with UK’s All-Party Parliamentary Groups
Source: PLoS One. 2021 Jun 24;16(6):e0252551. doi: 10.1371/journal.pone.0252551 (PMC8224875; doi:10.1371/journal.pone.0252551)
Supplement: S5 Table — (DOCX) [file pone.0252551.s005.docx]

## **S5 Table. Purpose of in-kind payments based on descriptions**

| Purpose of in-kind payment | Payments - n | Payments with value - n | Value of payments - £ | Payments involving pharmaceutical companies – n (%)* | Payments involving pharmaceutical companies with value – n (%)* | Value of payments from pharmaceutical companies - £ (%)* |
| --- | --- | --- | --- | --- | --- | --- |
| Secretariat or administrative support | 630 | 301 | 3,566,893.73 | 41 (6.51) | 20 (6.45) | 361,076.06 (10.12) |
| Membership fee | 60 | 60 | 547,392.48 | 32 (53.33) | 32 (53.33) | 292,175.57 (53.38) |
| Events (including receptions, meetings, conferences, awards) | 111 | 45 | 242,881.84 | 34 (30.63) | 30 (66.67) | 163,574.6 (67.35) |
| Inquiry costs | 6 | 6 | 108,744.04 | - | - | - |
| Report costs | 10 | 8 | 91,722.64 | 2 (20) | 2 (25) | 17,393.27 (18.96) |
| More than one purpose | 14 | 4 | 53,693.47 | 1 (7.14) | 1 (25) | 32,250.5 (60.06) |
| Translation or transcription | 7 | 7 | 32,169.17 | - | - | - |
| Travel and/or accommodation | 35 | 9 | 31,930.58 | - | - | - |
| APPG staff or advisor | 6 | 1 | 3,750.50 | - | - | - |
| No description (financial payments) | 298 | 298 | 2,604,236.449 | 58 (19.46) | 58 (19.46) | 344,875.81 (13.24) |
| Total | 1177 | 739 | 7,283,414.90 | 168 (14.27) | 143 (19.35) | 1,211,345.8 (16.63) |

*Percentages are the number/value of payments provided by pharmaceutical companies as a proportion of the total number/value of payments provided for each category by all donors
