# Supplementary material for: A hidden web of policy influence: The pharmaceutical industry’s engagement with UK’s All-Party Parliamentary Groups
Source: PLoS One. 2021 Jun 24;16(6):e0252551. doi: 10.1371/journal.pone.0252551 (PMC8224875; doi:10.1371/journal.pone.0252551)
Supplement: S6 Table — (DOCX) [file pone.0252551.s006.docx]

## **S6 Table. Payments from pharmaceutical industry-funded patient organisations**

| **Category and APPG name** | **Number of payments** | **Number of payments with value provided** | **Value of payments** | **Payments from pharmaceutical industry funded patient organisations – n (%)**** | **Payments with a value from pharmaceutical industry funded patient organisations – n (%)**** | **Value of payments from pharmaceutical industry funded patient organisations - £ (%)**** |
| --- | --- | --- | --- | --- | --- | --- |
| **Physical or mental health conditions** | **446** | **284** | **2,515,740.33** | **199 (44.62)** | **102 (35.92)** | **785,568.72 (31.23)** |
| Cancer* | 54 | 50 | 442,318.21 | 8 (14.81) | 4 (8) | 188,015.54 (42.51) |
| Sepsis | 11 | 8 | 154,979.29 | 3 (27.27) | 3 (37.50) | 71,441.14 (46.10) |
| Rare, Genetic and Undiagnosed Conditions | 4 | 4 | 65,595.93 | 4 (100) | 4 (100) | 65,595.93 (100) |
| Dementia | 17 | 13 | 64,296.82 | 17 (100) | 13 (100) | 64,296.82 (100) |
| Thrombosis* | 9 | 5 | 224,094.40 | 1 (11.11) | 1 (20) | 61,215.53 (27.32) |
| Autism | 8 | 4 | 45,065.61 | 8 (100) | 4 (100) | 45,065.61 (100) |
| Breast Cancer | 10 | 6 | 34,058.30 | 10 (100) | 6 (100) | 34,058.3 (100) |
| Pancreatic Cancer | 8 | 4 | 33,935.61 | 8 (100) | 4 (100) | 33,935.61 (100) |
| Brain Tumours | 10 | 4 | 46,494.61 | 10 (100) | 4 (100) | 30,997.96 (66.67) |
| Multiple Sclerosis | 12 | 4 | 29,344.54 | 12 (100) | 4 (100) | 29,344.54 (100) |
| Muscular Dystrophy | 9 | 5 | 19,250.08 | 9 (100) | 5 (100) | 19,250.08 (100) |
| Haemophilia and Contaminated Blood | 10 | 6 | 17,168.74 | 10 (100) | 6 (100) | 17,168.74 (100) |
| Osteoporosis | 10 | 3 | 16,188.16 | 10 (100) | 3 (100) | 16,188.16 (100) |
| Diabetes* | 11 | 7 | 20,575.70 | 9 (81.82) | 5 (71.43) | 13,074.7 (63.54) |
| Motor Neurone Disease | 8 | 4 | 10,693.15 | 8 (100) | 4 (100) | 10,693.15 (100) |
| Heart and Circulatory Diseases | 7 | 3 | 10,063.56 | 4 (57.14) | 3 (100) | 10,063.56 (100) |
| Ovarian Cancer | 8 | 4 | 9,300.58 | 8 (100) | 4 (100) | 9,300.58 (100) |
| Children, Teenagers, and Young Adults with Cancer | 4 | 4 | 9,106.27 | 4 (100) | 4 (100) | 9,106.27 (100) |
| Blood Cancer | 3 | 3 | 8,489.30 | 3 (100) | 3 (100) | 8,489.3 (100) |
| Inflammatory Bowel Disease | 2 | 2 | 7,674.77 | 2 (100) | 2 (100) | 7,674.77 (100) |
| Parkinson's | 12 | 3 | 6,938.67 | 12 (100) | 3 (100) | 6,938.67 (100) |
| Spinal Cord Injury | 8 | 4 | 20,454.20 | 7 (87.50) | 3 (100) | 6,938.67 (33.92) |
| Attention Deficit Hyperactive Disorder | 1 | 1 | 5,250.50 | 1 (100) | 1 (100) | 5,250.5 (100) |
| Allergy | 6 | 2 | 4,747.44 | 6 (100) | 2 (100) | 4,747.44 (100) |
| Epilepsy | 6 | 2 | 4,664.55 | 6 (100) | 2 (100) | 4,664.55 (100) |
| Atrial Fibrillation* | **8** | **4** | **51,935.62** | 1 (12.50) | 1 (25) | 3,750.5 (7.22) |
| Myalgic Encephalomyelitis (ME) | 12 | 4 | 9,494.88 | 6 (50) | 2 (50) | 3,554.68 (37.44) |
| Stroke | 5 | 1 | 2,385.53 | 5 (100) | 1 (100) | 2,385.53 (100) |
| Meningitis | 1 | 1 | 2,361.91 | 1 (100) | 1 (100) | 2,361.91 (100) |
| Cardiac Risk in the Young | 3 | - | - | 3 (100) | - | - |
| Cystic Fibrosis | 2 | - | - | 2 (100) | - | - |
| Headache Disorders | 5 | 1 | 5,565.53 | 1 (20) | - | - |
| **Medical specialty or specialist area** | **138** | **80** | **1,091,557.83** | **28 (20.29)** | **6 (7.50)** | **58,881.64 (5.39)** |
| Liver Health* | 10 | 6 | 59,906.00 | 7 (70) | 3 (50) | 30,062.41 (50.18) |
| Sexual and Reproductive Health* | 16 | 11 | 115,768.08 | 7 (43.75) | 3 (27.27) | 28,819.23 (25.89) |
| Eye Health and Visual Impairment* | 9 | 1 | 32,250.50 | 4 (44.44) | - | - |
| Kidney | 4 | - | - | 4 (100) | - | - |
| Respiratory Health | 6 | - | - | 6 (100) | - | - |
| **Social or mental wellbeing** | **118** | **55** | **656,443.19** | **23 (19.49)** | **9 (16.36)** | **48,364.28 (7.37)** |
| Baby Loss | 4 | 4 | 29,114.20 | 4 (100) | 4 (100) | 29,114.2 (100) |
| Young Disabled People | 8 | 4 | 15,499.58 | 4 (50) | 4 (100) | 15,499.58 (100) |
| Mental Health | 24 | 2 | 7,501.00 | 15 (62.50) | 1 (50) | 3,750.5 (50) |
| **Treatment, medical care, or patient support** | **88** | **43** | **559,425.37** | **24 (27.27)** | **8 (18.60)** | **35,675.4 (6.38)** |
| Stem Cell Transplantation | 10 | 6 | 26,552.76 | 10 (100) | 6 (100) | 26,552.76 (100) |
| Patient Safety | 1 | 1 | 5,372.13 | 1 (100) | 1 (100) | 5,372.13 (100) |
| Carers | 5 | 1 | 3,750.50 | 5 (100) | 1 (100) | 3,750.5 (100) |
| Maternity | 10 | - | - | 8 (80) | - | - |
| **Process of ageing or dying** | **35** | **16** | **85,505.03** | **15 (42.86)** | **7 (43.75)** | **31,860.79 (37.26)** |
| Ageing and Older People | 8 | 4 | 21,698.59 | 8 (100) | 4 (100) | 21,698.59 (100) |
| Hospice and End of Life Care | 8 | 4 | 18,534.33 | 6 (75) | 2 (50) | 7,911.7 (42.69) |
| Terminal Illness | 1 | 1 | 2,250.50 | 1 (100) | 1 (100) | 2,250.5 (100) |
| **Health of a collective** | **224** | **204** | **1,642,020.76** | **7 (3.13)** | **3 (1.47)** | **25,704.11 (1.57)** |
| Alcohol Harm* | 8 | 4 | 51,264.61 | 3 (37.50) | 2 (50) | 23,318.58 (45.49) |
| Men's Health | 4 | 1 | 2,385.53 | 4 (100) | 1 (100) | 2,385.53 (100) |
| **Medical research, prescribing pharmaceuticals, or the pharmaceutical industry specifically** | **53** | **11** | **303,554.86** | **8 (15.09)** | **-** | **-** |
| Medical Research | 32 | 4 | 139,091.19 | 8 (25) | - | - |
| Grand Total | 452 | 220 | 1,913,431.96 | 304 (67.26) | 132 (60) | 986,054.94 (51.53) |

*Those marked with an asterisk also received payments from the pharmaceutical industry

**Percentages are the number/value of payments from industry-funded patient organisations as a proportion of all payments received by each category (bold) or individual APPG
