# Supplementary material for: A hidden web of policy influence: The pharmaceutical industry’s engagement with UK’s All-Party Parliamentary Groups
Source: PLoS One. 2021 Jun 24;16(6):e0252551. doi: 10.1371/journal.pone.0252551 (PMC8224875; doi:10.1371/journal.pone.0252551)
Supplement: S7 Table — (DOCX) [file pone.0252551.s007.docx]

## **S7 Table. Categories of the in-kind payments provided by pharmaceutical industry-funded patient organisations**

| Purpose of in-kind payment | Payments - n | Payments with value - n | Value of payments - £ | Payments from pharmaceutical industry funded patient organisations - n | Payments from pharmaceutical industry funded patient organisations with value - n | Value of payments from pharmaceutical industry funded patient organisations - £ |
| --- | --- | --- | --- | --- | --- | --- |
| Secretariat or administrative support | 630 | 301 | 3,566,893.73 | 254 (40.32) | 117 (38.87) | 911,452.65 (25.55) |
| Membership fee | 60 | 60 | 547,392.48 | - | - | - |
| Events (including receptions, meetings, conferences, awards) | 111 | 45 | 242,881.84 | 30 (27.03) | 5 (11.11) | 17,019.41 (7.01) |
| Inquiry costs | 6 | 6 | 108,744.04 | - | - | - |
| Report costs | 10 | 8 | 91,722.64 | 5 (50) | 4 (50) | 19,075.55 (20.8) |
| More than one purpose | 14 | 4 | 53,693.47 | 7 (50) | 1 (25) | 5,565.53 (10.37) |
| Translation or transcription | 7 | 7 | 32,169.17 | 7 (100) | 7 (100) | 32,169.17 (100) |
| Travel and/or accommodation | 35 | 9 | 31,930.58 | 1 (2.86) | 1 (11.11) | 772.62 (2.42) |
| APPG staff or advisor | 6 | 1 | 3,750.50 | - | - | - |
| Total | 879 | 441 | 4,679,178.46 | 304 (34.58) | 135 (30.61) | 986,054.94 (21.07) |
