# Supplementary material for: A hidden web of policy influence: The pharmaceutical industry’s engagement with UK’s All-Party Parliamentary Groups
Source: PLoS One. 2021 Jun 24;16(6):e0252551. doi: 10.1371/journal.pone.0252551 (PMC8224875; doi:10.1371/journal.pone.0252551)
Supplement: S1 Appendix — (DOCX) [file pone.0252551.s008.docx]

## **S1 Appendix. Preparing the data for analysis**
