# Supplementary material for: A hidden web of policy influence: The pharmaceutical industry’s engagement with UK’s All-Party Parliamentary Groups
Source: PLoS One. 2021 Jun 24;16(6):e0252551. doi: 10.1371/journal.pone.0252551 (PMC8224875; doi:10.1371/journal.pone.0252551)
Supplement: Attachment — Submitted filename: Responses to reviewers.docx [file pone.0252551.s009.docx]

This document presents our responses to the comments received following the review of our manuscript. We first address the points made by the Editor, and then address the comments made by Reviewers 1 and 2, respectively. In addition to engaging with the below comments, we also refined our discussion section.

**Editor**

We would like to express our gratitude for the time you have taken to review our manuscript. We have enjoyed addressing and engaging with all of the insightful and thought-provoking comments that were kindly provided.

| **Editor’s comment** | **Authors’ response** |
| --- | --- |
| Are there Parliamentary rules or legislation governing the activities of APPGs? | We have introduced more detail about the regulations of APPGs in the introduction:  “APPGs are required to register with the Parliamentary Commissioner for Standards and maintain transparency through declaring payments from external donors. If consultancies or charities act as Secretariats they “must be prepared to disclose information” [16] about their clients or donors, respectively, either on their own websites or on request. However, with the exception of spending regulations on campaigning to support a particular party and prohibitions on paid advocacy specific to individual APPG members (rather than the APPG itself), they are subject to very few regulations regarding their activities or funding [16].” (lines 133-140, p. 3) |
| Has there been any research into how members of APPGs feel about accepting money from corporate interests? | No research has looked at how APPG members feel about corporate funding, however media news coverage has quoted MPs’ views on this matter. We have now included this in the Introduction, alongside referencing a recently launched Parliamentary inquiry which is looking into, amongst other things, corporate funding:  “Indeed, some Members of Parliament continue to question whether corporate funding should be allowed at all [25]. These problems are being investigated via an ongoing Parliamentary inquiry [26].” (lines 150-152, p.3) |
| An ethics statement is needed – either that it was obtained or that it was not necessary. | The study did not require ethical approval (as it draws on publicly available data at the organisational level), however it is part of a bigger project which has ethical approval from the University of Bath’s Social Sciences Research Ethics Committee (approval code: S19-073). |
| Line 106: Explain what is meant by "under the guise of Secretariats". | We have now rephrased this sentence: “In particular, lobbyists sometimes act as secretariats for APPGs to gain privileged informal access to legislators” (lines 149-150, p. 3)  On this point, we have also provided more information about what secretariats are in the main text which was previously only in a supplementary file: “They often have a ‘secretariat’, an organisation providing administrative support, to facilitate their functioning.” (lines 130-131, p. 3).  We also return to the point on secretariats to relate it to our findings in the discussion:  “Previous research has found patient organisations can feel pressured to align with industry agendas in ways that prioritise commercial over patient interests [53-55], which is concerning in the context of APPGs as the majority of industry-funded patient organisations’ payments were for secretariat purposes. Additionally, most of the pharmaceutical industry’s indirect payments were to fund third-parties to act as secretariat. These payments represent “infrastructural” conflicts of interests as secretariats help with the general running of APPGs, therefore holding a prime position and offering further scope to influence an APPG’s policy agenda.” (lines 456-553, p. 16) |
| Lines 185-186: Explain further what is meant by "whether industry was involved". | We have now added clarification about what we mean by industry involvement in publications:  “…indicated whether industry was involved financially (providing funding for the publication) or non-financially (such as providing a written contribution), and highlighted the key topics” (lines 383-385, p. 5). |

**Reviewer 1**

We would like to express our appreciation for the time you took to review our manuscript. We appreciate the insightful comments you have provided and have enjoyed engaging with them.

| **Reviewer 1’s comment** | **Authors’ response** |
| --- | --- |
| The authors have found an interesting and important case study to examine industry influence over policymaking. However, I had never heard of All-Party Parliamentary Groups and still do not have a fully clear idea of how they function, nor how these data might translate to other countries. In regard to the transferability of the findings, it would be useful to first position this work within the context of a literature on industry lobbying and its impacts.  Thus, I would suggest reorganizing the introduction so the reader is first introduced to the problem (lobbying/sponsorship, conflict of interest and its impacts on policymaking), and then to the particular case (explaining the political function of All-Party Parliamentary Groups). It would also be helpful to suggest whether there are analogous political bodies in other settings (if they exist). I would also clearly define All-Party Parliamentary Groups and their role in the abstract. My preference as a reader is to avoid acronyms at all costs. Much of this is currently in the Discussion, but should in part, be discussed up front. | We have now reorganised the Introduction so that we start by focusing on conflicts of interest and lobbying in relation to the pharmaceutical industry. The opening paragraph is now:  “Concerns have long been raised that wide-ranging financial ties to the pharmaceutical industry risk unduly influencing professional judgements [1]. These risks are prevalent in the context of *individual* conflicts of interest in scientific and policy decision-making, including payments to members of expert advisory panels [2, 3], scientific article authors [4], and physicians [5, 6], as well as *institutional* conflicts of interests through payments to seemingly independent third parties [7] such as patient organisations [8]. These widespread strategies form part of the pharmaceutical industry’s ‘web of influence’ [9, 10] seeking to shape the ideologies of the individuals and institutions they fund [11-13] to protect companies’ commercial interests, often at the expense of patient health outcomes [14].” (lines 115-123, p.3).  In the Introduction we have also now expanded the section which presents what APPGs are by incorporating the content of S1 Appendix. (lines 125-240, p. 3).  We now provide a definition of All-Party Parliamentary Groups in the Abstract:  “APPGs are informal cross-party groups revolving around a particular topic run by and for Members of the UK’s Houses of Commons and Lords. The facilitate engagement between parliamentarians and external organisations, providing expertise on complex policy matters, often through hosting events, and publishing reports.” (lines 53-56, p. 2)  Regarding your important point about bodies similar to APPGs in other contexts, we are not aware of any given the unique set-up (cross-party and informal) of APPGs. We have therefore added the following points in the introduction:  “To our knowledge, no bodies analogous to APPGs exist in other contexts, however previous research has documented how the pharmaceutical industry engages with other political landscapes, for example Parliament in Poland [13] and congress in the US [14, 27]. The majority of research, however, has prioritised drug companies’ ‘downstream’ lobbying tactics, targeting, in particular, expert advisory bodies or public payer institutions taking decisions on specific drug therapies [28-32]. With few exceptions [13], we know little about how the industry engages with the ‘upstream’ of the policy process, that is the bodies setting the ‘rules of the game’ for those at the ‘downstream’ level, an area frequently lobbied by the tobacco industry [33].” (lines 154-229, p. 3-4).  We also place more emphasis on the importance of recognising institutional conflicts of interests in the discussion:  “Previous research has found patient organisations can feel pressured to align with industry agendas in ways that prioritise commercial over patient interests [53-55], which is concerning in the context of APPGs as the majority of industry-funded patient organisations’ payments were for secretariat purposes. Additionally, most of the pharmaceutical industry’s indirect payments were to fund third-parties to act as secretariat. These payments represent “infrastructural” conflicts of interests as secretariats help with the general running of APPGs, therefore holding a prime position and offering further scope to influence an APPG’s policy agenda.” (lines 1546-1553, p. 16).  We have also removed acronyms throughout the text, with the exception of ‘APPG’ for All-Party Parliamentary Group given the frequency that this term is used. |
| The authors sought to provide evidence of “potential conflicts of interest” by identifying “all publications available on their websites and coded them by their type (e.g. report) and indicated whether industry was involved or whether the topics covered might be of interest to industry.” I first suggest eliminating mention of “potential” conflicts of interest throughout; a conflict of interest creates a risk for a primary obligation but it either exists or it doesn’t. However, I think the authors need to first clearly define what a conflict of interest is in the context of All-Party Parliamentary Groups (ie are these pertinent to individuals? Or the institution? What is the primary interest or obligation placed at risk?) to justify whether this actually measures the concept. For example, in section 3.5 why do these publications represent a conflict of interest? It could be that actually what is measured is industry influence, which I think is conceptually distinct from a conflict of interest and conflict of interest should not be used euphemistically to stand in for industry interference in policymaking. You get to this by the Discussion, but I think this should be right up front! | We have eliminated the use of the word ‘potential’ throughout the paper.  We appreciate your valuable suggestion that we need to clearly define what we mean by conflict of interest in the context of All-Party Parliamentary Groups. We now clarify this in the introduction:  “We explore payments received by health-related APPGs from pharmaceutical companies and industry-funded patient organisations between 2012 and 2018. We suggest that, in the context of health related APPGs, payments from the pharmaceutical industry represent institutional conflicts of interest as they create circumstances where the primary interest (policymaking in the interests of public health) is at risk of being unduly influenced by the secondary interest (the pharmaceutical industry’s goal of maximising profits).” (lines 231-246, p. 4).  Thank you for raising this point about whether the publications represent conflicts of interest. In the methods we now clarify that we examined the publications to contextualise our findings related to the payments and further show how industry might be involved in APPG activities:  “For the top five APPG recipients of pharmaceutical industry funding, we supplemented the information provided in the Register with information on their websites to exemplify the importance of APGPs for industry. We chose the top 5 as combined they received over three quarters of the total value provided by industry.” (lines 379-385, p. 5).  We have also strengthened the section on APPG outputs by suggesting that there may be a link between providing payments to APPGs and involvement in APPG activities:  “Nineteen of the 28 contributors had provided payments to the APPG publishing the report. This suggests that there is a link between providing payments to APPGs and being involved in their activities. It is also important that additional pharmaceutical companies were involved in APPG activities despite not providing any payments, suggesting that, in some instances, the involvement of the pharmaceutical industry extends beyond what is disclosed in the APPG Register.” (lines 1429-1434, p. 14) |
| The authors later make recommendations about transparency. However, they also had two public databases at their disposal. Perhaps there could be an added paragraph in the Intro or Methods that provides some context for these data – when/why was the Register created and what data does it include (include S3 Appendix)? Similarly, when/why were the pharma payments made public and what data does this database include? | Thank you for raising this important point. The section on Data Sources in the Methods now acknowledges the data sources in terms of being publicly available and improving transparency. However, it is also important to note that, although the data sources provide valuable information, we (the authors) had to manually create the databases as the information is currently not available to download as a dataset. We have emphasised the importance of these data sources being available to publicly access:  “We used two publicly available data sources, both of which have been made accessible to increase transparency and accountability of, separately, APPGs, patient organisations and drug companies.” (lines 259-261, p. 4)  We have also added a reflection on the user-friendliness of the data sources:  “Although these data are publicly accessible, consistent with findings from a content analysis of European disclosures of industry payments to healthcare professionals [39] they have limited usability. Therefore, we had to create Excel databases to facilitate analysis - we detail our approach to this below.” (lines 266-270, p. 4)  We also provide additional details about the content of each data source throughout the Methods. For the APPG data, we incorporated the content of S3 Appendix into the main text so that this information is available to the reader up front:  “All registered APPGs are required to report details of any support received from external sources – this can be a financial benefit or a benefit in kind - if the total value from that source exceeds £1,500 in the calendar year. Financial benefits (henceforth *financial payments*), monetary payments to the APPG, are disclosed with the name the donor and the payment value. Benefits in kind (henceforth *in-kind payment*s) involve providing goods or services to APPGs, for example funding events or membership fees, and are disclosed with the name of the donor and a brief description. Prior to July 2015, in-kind payments did not consistently have a payment value disclosed, but since July 2015 in-kind payments have been disclosed with approximate value in bands of £1,500 (e.g. £15,001-16,500). In these cases, bracket averages were calculated as the approximate value (e.g. £15,751). In the results we at times distinguish between financial and in-kind payments as they are subject to different reporting requirements (namely the payment descriptions). As payment values are not always provided for data from the archived Register, we consistently provide the number of payments alongside values.  Separately, payments can be *direct* (donor provides payment to an APPG) or *indirect* (one or more donor funds a third-party to provide a payment to an APPG). As an indirect payment can be funded by multiple donors, we refer to these as ‘contributions’ throughout the results section to capture that one indirect payment can be funded by multiple organisations.” (lines 459-478, p. 6).  For the patient organisations, we added some additional information about the content of the data, without going into too much detail as the methods are described in another open-access paper (referenced in text):  “Briefly, the database contains pharmaceutical company donors, patient organisation recipients, payment dates, descriptions, and values.” (lines 454-455, p. 6). |
| It would be helpful at the beginning of the Methods to have a small sub-section outlining the overall study design and population from which you sampled. | We have added a brief summary of our study design and population in the first paragraph of the Methods.  “This section explains the process of data collection and analysis for our cross-sectional study focusing on 146 health-related APPGs” (lines 255-256, p. 4). |
| The Results are difficult to follow. The text is quite dense and the key points are often lost. I would suggest ensuring that no information in the Tables is duplicated in the text. Then, to clearly highlight the key finding for each section. By section 3.3 it is becoming rather repetitive and it is unclear what the added value of parsing the data in these different ways adds. We need more of the author’s analysis to interpret these data, explaining to the reader why it is important, for example, to understand the total impact of relationships #1 and 2 versus these separately, versus payments overall. | We appreciate your insight into how the Results section could be streamlined. We have gone through the results section very closely and removed any unnecessary information (i.e. that which is already presented in the tables). We have also removed the two subsections for donors and recipients within direct payments and indirect payments. This has made the results less dense and repetitive, and the focus now is on the key points within each of the three relationships.  We have also introduced more interpretation of the differences between relationships #1 and #2. For example:  “Different companies prioritised direct and indirect payments. Overall, industry provided more direct payments than indirect.” (lines 903-905, p. 10).  “Industry’s indirect payments targeted nine health-related APPGs from the same three categories as their direct payments” (lines 907-909, p. 10).  “Similar to the targeted funding identified in the direct payments, over half of industry’s indirect payments went to two APPGs (although these were different - Sickle Cell and Thalassaemia and Thrombosis).” (lines 911-914, p. 10).  The results section is now structured as follows:   - Overview of all payments - Direct payments from pharmaceutical companies (presented as one section rather than two as before) - Industry payments from pharmaceutical companies (presented as one section rather than two as before) - Direct and indirect payments combined (to provide a summary of all payments from pharmaceutical companies) - Payments from industry-funded patient organisations - APPG outputs / publications |
| There is a lot of descriptive information here, but I think the paper could be strengthened by some comparative analysis. For example, you separately present “direct payments” from the recipient and donor perspective, but the interesting part would be whether this is concordant or discordant. Could you instead triangulate these two data sets and present this information together? What can we learn about direct payments, for example, taking these two data sources together? The numerous supplementary tables that present these data separately (I think?) do not add anything to the analysis; thus, they should be framed as data availability/shared data, or should be further analysed to triangulate the data sources. | We have now amended the results section so that payments from the donor and recipient perspective are reported together, whilst also removing excessive descriptive information.  This has allowed us to draw more on similarities between the donor and recipient data:  “Concentration was noted in recipients as well as donors, with industry payments comprising at least 40% of the direct income received by half of the APPGs reporting industry payments.” (lines 823-825, p. 9).  “As with direct payments, indirect payments were concentrated by donors, with the top three drug company donors providing over half of the industry’s indirect payments (see S2 Table for a list of companies).” (lines 901-903, p. 10).  With regards to the supplementary tables, we feel that these provide important background figures to the data we present in the text. We did not want to overwhelm the reader with these data in the main text, but in the spirit of open data we wanted them to be easily accessible to an interested reader alongside the open access dataset. We have, however, reduced the number of supplementary tables by removing the table of other donor types (as this detracts from our main message of the pharmaceutical industry’s financial involvement in APPGs). One table was also duplicated, which we have now removed.  Importantly, to reduce reader burden we have also reduced the number of Appendices from 5 to 1 by incorporating their contents into the introduction and methods sections. We feel that this now makes the number of supplementary tables more digestible.  In-text and in the supplementary tables we have removed median values and interquartile ranges. We felt that this added unnecessary complexity and reader burden without actually adding to the story. |
| Figure 2 provides a nice typology of payment types and can guide the reader through the presentation of the results. However, I would save some of the theorising for the discussion (mechanisms of influence) and just clearly show the different kinds of payments (leaving aside the ‘potential COI’ etc). Then, the results can present: direct financial payments from pharma to Group, indirect payments through third-party, and payments from pharma-funded patient group. My main question regarding this typology is whether the 3rd category (payments from pharma-funded patient groups) is actually a sub-category of the indirect payments? If not, what is the distinction? | Thank you for your positive words about how we categorise the payment types. We now refer to it as a typology in text!  Importantly, the three elements in the typology of payments are distinct, therefore we have now clarified the differences between them to reflect this. Below is the new text which seeks to make the distinction between relationships 2 and 3 by further explaining what each relationship is, as well as clearly specifying the different data sources which inform the analysis of each relationship to further signify their differences:  “In addition, Relationship #2 involves conflicts of interests through indirect payments, that is organisations being funded by pharmaceutical companies with the specific purpose of providing a service for an APPG, as disclosed in the APPG Register. Finally, Relationship #3 covers payments from patient organisations, as disclosed in the APPG Register, which have coincidentally received payments from the pharmaceutical industry (identified in drug company disclosures of payments to patient organisations).” (lines 586-591, p. 7). |
| The numerous supplementary files include a great deal of material which is essential to understand the case study (e.g. S1 Appendix – this should just be in the introduction; S3 Appendix is necessary to understand the data set – this should be in the intro or methods per my comment above) and also to evaluate the rigour of the methods (the two figures). Having this many supplementary files also creates a great deal of reviewer/reader burden. I suggest that the authors carefully consider what is indeed supplementary and incorporate more of this material into the manuscript (and consider what is essential), or eliminate it. For example S2 Appendix – these terms should be defined in the text, or the authors should use a limited number of these terms to avoid confusion and reduce the need for a glossary. The Supplementary Tables including data seem more appropriate as true supplementary files. | We have now incorporated S1 Appendix (additional information about APPGs) into the introduction (lines 130-133, p. 3). We agree that this is crucial information which should be presented up front to allow the reader to fully understand what an APPG is without having to seek out the supplementary file  We have incorporated the content of S2 Appendix (Glossary of key terms), so that all the necessary definitions of terminology used in the APPG Register are discussed in the main text (lines 459-478, p. 6). We agree that these definitions are important to be presented and explained up front to guide the reader through the paper’s findings.  We have also now incorporated S3 Appendix (details about what is included in Register entries) into the methods alongside the contents of S2 Appendix to ensure that all details necessary for understanding our paper are provided to the reader (lines 459-478, p. 6). On reflection, we have also removed S4 Appendix (identifying registered APPGs) as this mainly repeats what is already explained in-text and in the footnotes for what was S5 Appendix (now S1 Appendix).  Therefore, we have reduced the number of Appendices from 5 to 1. |
| Table S1 (categories of donors) – it seems that the iterative categorisation is not quite finished. This perhaps could be more meaningfully combined into fewer categories. I think this should be guided by a research question and/or framework – i.e. what categories of sponsor are concerning? | Thank you for drawing our attention to the categorisation of other donors. After careful consideration, we have decided to remove the table and accompanying sentence in the main text which refer to the categorisation of donors. We feel that it detracts too much from the main message of the paper (the extent of industry funding) and complicates the findings more than they need to be. We agree that other categories of donors may be important and future research could explore this data further with a focus on other donors. We also made this decision to reflect comments from both reviewers about the complexity of the results section. |
| Minor comments |  |
| The Methods would be much clearer on first read with the aid of Supplementary Figures 1 and 2 – could these not just be part of the main text (ie not supplementary)? Suggest editing SF2 to eliminate “potential” from conflict of interest. | We agree that these would figures are integral to the main text given the important context for the data they provide. We have now reuploaded the figures as Fig1 and Fig2 (rather than S1 Fig and S2 Fig).  We confirm we have removed the word ‘potential’ throughout the text and figures. |
| In the first line of the abstract and in the introduction you mention both “conflicts” and “coincidences” of interests. I am intrigued by this phrasing, but because it is so conceptually interesting, I would appreciate a sentence or two of background, which include definitions of how you conceptualise and differentiate these terms. This is important in both the abstract and main text. | Due to the comments kindly provided by Reviewer 2, and the consistent comments from both reviewers about the unnecessary complexity of how our findings were presented, we have removed the term ‘coincidences of interest’ from the paper. We instead refer to these as payments from patient organisations with conflicts of interest. |
| The abstract findings need more contextualising – 16 All-Party Parliamentary Groups received pharma funding, but out of how many in total? Of the sponsoring patient groups, out of how many? | We have now added additional values to the abstract to give the important and necessary context to the percentage values. Namely, we added: overall figures for total number of health related APPGs, total value of payments from all donors, total number of payments from all donors, and total number of patient organisations. |
| What is the reason for beginning data collection in 2012? | We have now added the following sentence at the start of section 2.1. to explain why we used 2012. “Secondly, drug company disclosure reports of payments to patient organisations which were made mandatory in 2012. We have complete data for drug company payment disclosure reports for 2012-2018, and therefore consider payments registered by APPGs between 2012-2018.” (lines 263-270, p. 4). |
| The proportions throughout the text could be rounded to whole numbers or just one decimal place for readability. | We have now expressed all in-text percentages with one decimal place. |
| Table 1 missing a (%) in the column header “Number of Payments with value – n” | We have added the missing (%). |
| Table 1; you state in the text that Cancer received the highest number of payments, but this is specific to payments from pharmaceutical industry sources. Could you flip the columns and present these on the left hand and the “payments from all external sources” on the right – it would be nice to have an additional column that gives the proportion of pharma payments out of the total received. | Thank you for these comments. We have now clarified that the APPG Cancer received the most payments from pharmaceutical companies and industry-funded organisations (lines 599-600, p. 7).  We have also amended the in-text commentary of Table 1 to reduce confusion around what the focus is. It now focuses solely on the combination of the 3 relationships (payments from pharmaceutical companies – direct and direct – and payments from industry-funded patient organisations). Previously, it mentioned payments from pharmaceutical companies as well as providing combined totals of the three relationships. The remainder of the results is where these relationships are broken down and explored further, whereas the opening section of the results now reports overall totals only.  We think that this now removes any confusion around Table 1 and that the ordering of the columns now works – first the table introduces the total value and number of payments received by APPGs from all sources, and secondly it shows the proportion of payments (number and value) from the pharmaceutical industry and industry-funded patient organisations. |
| The text frequently cross-references itself forcing the reader to jump around, e.g “these APPGs fell into three categories (introduced in Fig 1, reason for inclusion)”; it would enhance readability to just clearly present the necessary information at each point. | We have now removed the reference to Fig 1 and stated the three categories to ensure the reader follows the story and does not have to shift back and forth. |
| Table 2- what is the significance of the bolded text? Are these actually headers? Could this be formatted more clearly? | The bolded text is the category of APPGs and the standard text is the APPG name. We appreciate that this was not clear, so we have added additional information in the column heading and the table footnotes to show that the bold text represents categories. |

**Reviewer 2**

We would like to thank you for your extremely thoughtful and helpful comments on our manuscript. We appreciate you taking the time to review our work and we have endeavoured to address and engage with all points raised.

| **Reviewer 1’s comment** | **Authors’ response** |
| --- | --- |
| I definitely would like to see this paper published as it clearly illustrates the potential for the pharmaceutical industry to influence health policy through payments to groups that communicate directly with government, as well as patient organizations. This is a very important topic. However, I think this main message is obscured by all the confusing terminology in the paper, such as “potential” conflicts of interest, “conflicts of coincidence,” “indirect” and “direct” payments, “in-kind” payments, and “relationships” 1, 2, and 3. This paper clearly seems to be talking about financial payments and conflicts of interest. I would not make it more complicated than it needs to be. Also, not all of these terms are used as they commonly are in the COI literature.  My major suggestion is to eliminate all of these terms and just present the data with clear labels as in Table 1 (ie, payments from all external sources, payments from pharmaceutical companies, etc.) and indicate that these payments are a conflict of interest. I provide more specific comments on these terms below. | Thank you for your positive appraisal of our work. It means a lot that you can see its value.  We appreciate your feedback about the terminology used in the paper and have considered it extensively. We have removed the word ‘potential’ throughout the paper as we agree that it is unnecessary.  We have also removed the term ‘coincidences of interest’. We agree that introducing our different interpretation of this term adds unnecessary confusion and detracts from the paper’s main message.  We have not removed all terminology as we feel it has an important role in the overall message and presentation of our findings. However, we have made substantial changes to try to address your welcomed comments to ensure that the terminology adds to rather than detracts from our overall message. We have streamlined the results substantially and added additional details to the methods section about the different payment types disclosed in the APPG Register (lines 459-478, p. 6). We have also added more content relating to conflicts of interest as they relate to APPGs in the introduction (lines 232-251, p. 4) and discussion (lines 1447-1454, p. 16; lines 1560-1564, p. 17) which we feel is now the bigger focus. Therefore, we think the terminology used is now appropriate and no longer confuses but rather adds to the overall message. We provide specific rationale for retaining these terms below:   - ‘Financial’ and ‘in-kind’ payments   We believe this is an important distinction to acknowledge in the text in the name of transparency as this is how the payments are disclosed in the Register. However, we now place less emphasis on the distinction between financial and in-kind payments, only drawing on it when presenting the payment descriptions as payment descriptions are provided for in-kind payments only. We have removed it from Fig 2, the typology of relationships. We feel that it is still important to discuss this distinction in the methods section as in-kind payments are subject to different disclosure requirements than financial payments. We now explain this in the methods:  “In the results we at times distinguish between financial and in-kind payments as they are subject to different reporting requirements (namely the payment descriptions).” (lines 468-470, p. 6)   - ‘Direct’ and ‘indirect’ payments   These terms provide an important distinction between two ways in which the pharmaceutical industry can be financially involved with all-party parliamentary groups. While direct payments denote direct involvement in the form of a payment from the company to the APPG, the indirect payments suggest involvement at arm’s length. For this reason, we wanted present these payments transparently for what they are. We have added additional detail in the methods (lines 474-478, p. 6) about what direct and indirect payments are in the context of the APPG Register so that these terms do not appear out of nowhere in the results.   - Relationships 1, 2, and 3   We use this approach to describing the types of involvement the pharmaceutical industry can have with APPGs to show that they can be involved in various ways. We now incorporate the Figure presenting the typology in the main text to make the distinction between the types of relationships clearer. We have also clarified how these types of relationships are distinct (lines 595-600, p. 7). |
| Abstract |  |
| Need to define, in lay terms, what All-Party Parliamentary Groups are. Even in the abstract, if possible. Can they be summarized as lobbying groups? Try to describe the objectives of these groups in the abstract because context is needed to determine if there is a conflict of interest. In the introduction, the key description is in the first para: “they facilitate engagement between parliamentarians and external organisations, providing expertise on complex policy matters.” This does sound to me what would commonly be called a lobbying group, and this is enforced by the later comments in the Introduction regarding lobbyists acting under the disguise of Secretariats. | We have added a definition in the abstract:  “APPGs are informal cross-party groups revolving around a particular topic run by and for Members of the UK’s Houses of Commons and Lords. They facilitate engagement between parliamentarians and external organisations, disseminate knowledge, and generate debate through meetings, publications, and events.” (lines 53-56, p. 2)  APPGs do not refer to themselves as lobbying groups, therefore we a wary of describing them in this way. They are quite unique in that they are cross-party and informal, despite being based in Parliament. We now provide additional detail about what APPGs are in the introduction:  “They facilitate engagement between parliamentarians and external organisations, providing expertise on complex policy matters, hosting events, and publishing outputs including reports and inquiries. They often have a ‘secretariat’, an organisation providing administrative support, to facilitate their functioning. APPGs do not receive any funding from Parliament, but they can choose to accept payments to cover costs of events, secretariats, travel, reports, and other activities. APPGs are required to register with the Parliamentary Commissioner for Standards and maintain transparency through declaring payments from external donors. If consultancies or charities act as Secretariats they “must be prepared to disclose information” [16] about their clients or donors, respectively, either on their own websites or on request. However, with the exception of spending regulations on campaigning to support a particular party and prohibitions on paid advocacy specific to individual APPG members (rather than the APPG itself), they are subject to very few regulations regarding their activities or funding [16].” (lines 125-140, p. 3).  We have also explained that no similar bodies exist, to our knowledge, but that industry engagement with other political contexts internationally has been minimal, with a few exceptions:  “To our knowledge, no bodies analogous to APPGs exist in other contexts, however previous research has documented how the pharmaceutical industry engages with other political landscapes, for example Parliament in Poland [13] and congress in the US [14, 27].” (lines 154-157, p. 3). |
| “Fifty APPGs also had coincidences of interest, indicated by reporting 304 payments worth £986,054.94 from 57 patient organisations which had received £27,883,556.3 from pharmaceutical companies across the same period.” It is not clear what coincidence of interest means in this sentence. It’s a COI, patient groups supported by pharma who then support the APPGs. You could say it is an indirect payment that results in the COI. | We have removed the term ‘coincidences of interest’ throughout our paper. We now specify that what we are exploring are payments from patient organisations with conflicts of interest (indicated by their receipt of payments from the pharmaceutical industry).  We have revised the sentence as follows:  “In addition to the pharmaceutical industry’s payments, the APPG Register reported 50 APPGs receiving 304 payments (all in-kind) worth £986,055.0 from 57 patient organisations which feature as payment recipients in pharmaceutical industry payment disclosures.” (lines 1171-1173, p. 12). |
| Introduction |  |
| “Coincidence of interest” is defined in the introduction (line 126) as: “occurring when “a player crafts an array of overlapping roles across organisations to serve his own agenda-or that of his network-above that of the organisations which he works” [24]. In our study, the ‘player’ is the pharmaceutical company and the ‘overlapping roles’ are the financial ties to patient organisations involved in the health policy landscape via APPGs.” I would not classify financial ties as “overlapping roles.” They are simply payments which are a conflict of interest. I would think of “overlapping roles” as something more insidious, such as pharma companies and patient organizations having the same board members, or a former pharma employee founding a patient organization. It is my understanding that these types of nonfinancial relationships are beyond the scope of this paper.  Financial ties between pharmaceutical companies and patient groups have been extensively investigated. Based on this literature, financial payments to patient groups would clearly fit the definition of a conflict of interest. | We appreciate your comments on our use of the term ‘coincidences of interest’. To avoid any confusion, as mentioned in our responses above, we have now removed this term throughout the paper. Following your feedback, we now describe the coincidences of interest as patient organisations with conflicts of interest. We agree that this is a clearer way of describing this relationship.  In the introduction, we also now start by introducing conflicts of interests in the context of the pharmaceutical industry to set the tone of the paper going forward:  “Concerns have long been raised that wide-ranging financial ties to the pharmaceutical industry risk unduly influencing professional judgements [1]. These risks are prevalent in the context of *individual* conflicts of interest in scientific and policy decision-making, including payments to members of expert advisory panels [2, 3], scientific article authors [4], and physicians [5, 6], as well as *institutional* conflicts of interests through payments to seemingly independent third parties [7] such as patient organisations [8]. These widespread strategies form part of the pharmaceutical industry’s ‘web of influence’ [9, 10] seeking to shape the ideologies of the individuals and institutions they fund [11-13] to protect companies’ commercial interests, often at the expense of patient health outcomes [14].” (lines 115-123, p. 3). |
| Methods |  |
| Line 185: “we identified all publications available on their websites and coded them by their type (e.g. report) and indicated whether industry was involved or whether the topics covered might be of interest to industry.” The APPGs cover a wide variety of topics (Fig 1), so this is a very important classification. More information is needed on how the authors determined how “industry was involved” or “how the topics covered might be of interest to industry” | Thank you for raising this point. We have now amended the wording to provide more information about industry involvement and identifying topics:  “For the top five APPG recipients of pharmaceutical industry funding, we supplemented the information provided in the Register with information on their websites to exemplify the importance of APGPs for industry. We chose the top 5 as combined they received over three quarters of the total value provided by industry. We identified all publications available on their websites and coded them by their type (e.g. report), indicated whether industry was involved financially (providing funding for the publication) or non-financially (such as providing a written contribution), and extracted the key topics which were the focus of the publications.” (lines 379-386, p. 5). |
| Please explain the overlap in dates of data collection for the topics of the APPG (Which seem to be in 2020, according to lines 180-181) and the payments from pharmaceutical companies, which seem to be much earlier (2012-2018, according to lines 188-193). Should these be more closely aligned? | We collected the drug company disclosure data first as part of a separate project.  Importantly, we used the same dates (2012-2018) for both data sources. Therefore, although the data were collected at different times, this would not make a difference to years to which the data collected apply.  We have added additional clarity around the dates in section 2.1:  “Firstly, the UK Parliament’s Register of All-Parliamentary Groups (henceforth the APPG Register) which was first introduced in 2010, with guidelines to increase transparency published in 2015 and updated in 2017. Secondly, drug company disclosure reports of payments to patient organisations which were made mandatory in 2012. We have complete data for drug company payment disclosure reports for 2012-2018, and therefore consider payments registered by APPGs between 2012-2018.” (lines 261-266, p. 4) |
| I did not see the methods describing how patient organization and pharmaceutical company payments to APPGs were determined. Is this in the Register described in lines 151-162? Since payments to the APPGs are a main variable in this paper, the methods for obtaining this information should be clearly described in the body of the text. Lines 207-216 describe the types of payments recorded in the Register and this information should come earlier. | We now clarify that, firstly, all payments from all donors were extracted into a single database:  “ER extracted all available information on payments provided by all donors from the active (in Jan-March 2020) and archived (in April-May 2020) Registers into an Excel database.” (lines 348-350, p. 5).  We then note that we had to conduct web searches of all donors providing payments to APPGs to identify pharmaceutical companies and patient organisations to facilitate our analysis:  “We used web searches to identify pharmaceutical companies and patient organisations amongst the donors to identify our final sample of payments. Patient organisations were looked up in the database of pharmaceutical industry payments to patient organisations, and if there was a match the payment details were extracted into a separate Excel spreadsheet.” (lines 485-489, p. 6). |
| Line 197: It is not clear which database was being screened to eliminate duplicates – the database of payments to APPGs or the database of payments made by pharmaceutical companies? | We have added additional information to clarify that we are referring to the database of APPG payments. |
| The classification of direct and indirect payments (lines 222-224) is not what is commonly used in the conflict of interest literature. “Direct” payments usually refer to payments made directly to the person or organization of interest (eg, pharma pays the APPG). “Indirect” payments usually refer to payments that go through a 3rd party (eg, pharma pays a professional society that then pays an APPG). The definition used here: “direct payments involve one single donor, whereas indirect payments involve at least one other organization” needs clarification. It seems that what is really being calculated is the proportion of payments from a pharmaceutical company when there are payments from multiple organizations. Furthermore, this definition of “indirect” payments does not seem to correspond with the one in S3 which provides details of information in the APPG registries. In S3 states: “APPGs must also name the donor (and any third-party behind the funds if it is indirect)” | Thank you for drawing our attention to the confusing definitions we currently apply to direct and indirect payments. The definitions of direct and indirect which you provide represent the way we interpret these terms. We have therefore added additional clarity about the difference between direct and indirect payments and how this relates to the APPG data. Below is how we introduce these terms:  “Separately, payments can be *direct* (donor provides payment to an APPG) or *indirect* (one or more donor funds a third-party to provide a payment to an APPG). As an indirect payment can be funded by multiple donors, we refer to these as ‘contributions’ throughout the results section to capture that one indirect payment can be funded by multiple organisations.” (lines 475-483, p. 6). |
| Results |  |
| Line 244. Provide the denominator for the APPGs. It is not clear how many were actually included in the study. Also, it appears that 2 denominators are used in this paragraph – all health related APPGs and all APPGs reporting payments. | We have now ensured that all denominators are consistent and made clear. |
| Lines 247 and 249. Again, I find the use of “direct” and “indirect” confusing. Doesn’t the figure for indirect payments mean the proportion of payments from pharmaceutical companies if the payments were from multiple sources? The important piece of information to know is how many and what is the value of payments from pharmaceutical companies to APPGs. I think this is what is meant by “Relationships #1 and #2” in line 263. | Thank you for raising this points. We now start the Results section with one sentence summarising all payments from all donors to provide a point of comparison for the payments from pharmaceutical companies which are discussed throughout the remainder of the results section:  “In total, 120 of 146 (82.2%) health-related APPGs reported 1,177 payments from all external donors, with a total value of £7,283,414.90.” (lines 581-582, p. 7).  We have streamlined the opening section (lines 581-593, p. 7) of the results in the following ways:   - Shortened the opening paragraph which provides a concise summary of the contents of the APPG Register as a whole - Revised the next paragraph to clarify how the three types of relationships are understood, including the data sources used - Ensured that the presentation of the summary of the findings of the three types of relationship are presented clearly and concisely through:   - Clearly stating that the findings being presented relate to the three types of relationships   - Removing excessive repetition of the contents of the first table |
| Lines 255 – 259. Again, “indirect” and “coincidences” of interest are confusing. How do organizations being funded by pharmaceutical companies to provide a service for an APPG differ from consumer organizations that are funded by pharmaceutical companies and provide payments to APPGs? | We have now added some additional sentences which provide further context to the difference between the indirect payments and the payments from industry-funded patient organisations (previously coincidences of interest). For clarity, in-text we have also added which data source was used for each type of payment (direct, indirect, industry-funded patient organisations) to further display how these types of payments differ. The paragraph now reads:  “Relationship #1 relates to conflicts of interest through payments directly provided by a pharmaceutical company to an APPG, as disclosed in the APPG Register. In addition, Relationship #2 involves conflicts of interests through indirect payments, that is organisations being funded by pharmaceutical companies with the specific purpose of providing a service for an APPG, as disclosed in the APPG Register. Finally, Relationship #3 covers payments from patient organisations, as disclosed in the APPG Register, which have coincidentally received payments from the pharmaceutical industry (identified in drug company disclosures of payments to patient organisations).” (lines 586-593, p. 7) |
| Line 259: refers to “pharmaceutical industry funded organizations.” Do we know if the groups are completely funded by pharma or partially funded? This was not clear from the methods. | Within the scope of our study we have not compared the value of payments received by patient organisations with their total income. However, in light of the insightful reviewer feedback we have introduced a new table which shows the top 10 patient organisation donors to APPGs (by the value of payments provided) alongside the number and value of payments they received from the pharmaceutical industry (p. 12-13). |
| Table 1 is informative because it clearly states in the column headings where the payments are coming from. However, need to define how the percents are calculated. I believe these are row percents – which clearly shows that the Breast Cancer and Pancreatic Cancer APPGs are 100% funded by the pharmaceutical industry or pharma-supported patient groups, correct? | Yes, this is the correct interpretation of the percentages. We have now introduced an additional note under this table to clarify what the percentages represent:  “¶ Percentages are the number/value of payments from pharmaceutical companies and pharmaceutical industry-funded patient organisations as a proportion of the total number/value of payments received by each APPG” (lines 800-802, p. 9) |
| Please always provide denominators whenever percents are reported in the Results section. | We have now ensured that all denominators are provided. |
| Line 314-315. Mentions “in-kind payments.” These have not been previously defined, please clarify what this means in the Methods section. In-kind payments are mentioned in S2 and S3, but this is not sufficient as this does not explain if they are reported separately or combined with the other payments. | This is a very important point which we appreciate you picking up on. We have now incorporated, with minor adjustments and clarifications, the content of S2 into the main text. The Methods now includes the following paragraph which explains more about the types or payments and how they are reported by APPGs:  “Benefits in kind (henceforth *in-kind payment*s) involve providing goods or services to APPGs, for example funding events or membership fees, and are disclosed with the name of the donor and a brief description. Prior to July 2015, in-kind payments did not consistently have a payment value disclosed, but since July 2015 in-kind payments have been disclosed with approximate value in bands of £1,500 (e.g. £15,001-16,500). In these cases, bracket averages were calculated as the approximate value (e.g. £15,751). In the results we at times distinguish between financial and in-kind payments as they are subject to different reporting requirements (namely the payment descriptions). As payment values are not always provided for data from the archived Register, we consistently provide the number of payments alongside values.” (lines 464-473, p. 6). |
| Line 350-51 also mentions “in-kind payments” and gives the example of providing secretariat or administrative services. If no £ values were reported, how were the in-kind services counted? If information on the amount of payment is available, it would be clearer just to report the £ amounts and how the payments were used. My confusion regarding in-kind payments continues throughout the Results section. | We have now clarified in the Methods that: “Where values are not reported, we report the number of payments only”.  We now place less emphasis on differentiating between financial and in-kind payments. We have retained the distinction only where it is required in the findings to provide the number and value of payments with payment descriptions (as only in-kind payments are disclosed with payment descriptions).  We have also added the value of payments alongside the number of payments following your comment, and focus more on the key messages, in both the direct and indirect sections:  “Seventy-one industry payments worth £513,772.1 were in-kind and therefore disclosed with a description. The most frequent purpose for industry payments was events (33 payments worth £292,175.6) followed closely by membership fees (32 payments worth £292,175.6). The remaining six payments covered four secretariat costs (totalling £32,146.2), one report costs (£8,250.5) and one had two purposes – inquiry staff and event costs (£32.250.5).” (lines 889-893, p. 10).  “All of the indirect payments were in-kind and mainly covered funding third parties to provide secretariat or administrative services (37 payments with a disclosed value of £328,929.8), with the remaining two payments covering costs of a report (£9,142.8) and an event (£14,625.3). The pharmaceutical industry was a prominent funder at the infrastructural level, providing funding for 37 of the 65 (58.7%) indirectly funded secretariats.” (lines 924-1057, p. 10-11). |
| Only the APPGs are named in the results section. Why not name the pharmaceutical companies and patient organizations, at least those who are providing the most payments? The pharmaceutical company names are in Table S2 and S3, but it would be useful to at least have the top 10 in the text, especially in relation to the comment below. I did not see the patient organizations named in any supplemental tables. | We have now introduced two new tables into the Results section. Firstly, a table of the top 10 pharmaceutical company donors (by value of payments) on page 12. We have also added a section in the discussion linking the top 10 donors to those identified in previous research exploring industry payments to other organisations (lines 1472-1447, p. 16-17). Secondly, a table of the top 10 industry-funded patient organisations (by value of payments) alongside the number and value of payments they received from the pharmaceutical industry – on page 12-13. |
| The section on APPG outputs is important and interesting. But, as per previous comment, it would be useful to know that when the activities which involved drug companies, that these same companies were funding the APPGs (or not). This obviously would not establish any causality, but it would show the financial link between funders of the APPGs and the activities of the APPGs. | Thank you for your kind comments regarding the APPG outputs section. We appreciate your suggestion and have added these additional details.  “Nineteen of the 28 contributors had provided payments to the APPG publishing the report. This suggests that there is a link between providing payments to APPGs and being involved in their activities. It is also important that additional pharmaceutical companies were involved in APPG activities despite not providing any payments, suggesting that, in some instances, the involvement of the pharmaceutical industry extends beyond what is disclosed in the APPG Register.” (lines 1300-1305, p. 14). |
| Discussion |  |
| Line 562 states: “the concentration of payments within a narrow group of donors.” I did not see the list of donors in the Results section. | As mentioned in response to the previous comment about naming donors, we have now introduced a table with a list of the top 10 donors (by value of payments) in the Results in the section summarising the total payments from the pharmaceutical industry. We have also added a section in the discussion linking the top 10 donors to those identified in previous research exploring industry payments to other organisations (lines 1472-1447, p. 16-17). |
| Minor points |  |
| Data access: Clarify if the Bath Archive makes the data publicly available. Is this open access? | Yes, the Bath Archive makes the data publicly available and the data will be published open access. |
| Reference #28 contains extraneous information. | Thank you for pointing this out, we have now amended the reference. |
